# On-chip topological edge state cavities

**DOI:** 10.1038/s41377-025-02017-3

**Published:** 2025-09-18

**Authors:** Wenhao Wang, Zhonglei Shen, Yi Ji Tan, Kaiji Chen, Ranjan Singh

**Affiliations:** 1 Division of Physics and Applied Physics, School of Physical and Mathematical Sciences, Nanyang Technological University, Singapore, Singapore; 2 Centre for Disruptive Photonic Technologies, The Photonics Institute, Nanyang Technological University, Singapore, Singapore; 3 Department of Electrical Engineering, University of Notre Dame, Notre Dame, IN USA

**Keywords:** Terahertz optics, Micro-optics

## Abstract

Confining light in an on-chip photonic cavity with strong light-matter interactions is pivotal for numerous applications in optical and quantum sciences. Recently, topological valley photonics has introduced new schemes for light confinement with topological protection, enabling robust on-chip light manipulation. Here, we present a topological edge state cavity that confines light within a topological bandgap while robustly guiding it to circulate around the cavity via topological edge states. We demonstrate a giant enhancement in the intrinsic quality factor by three orders of magnitude, while simultaneously increasing the free spectral range from 5.1 to 7.1 GHz through tailoring the radiation leakage and group index of topological valley edge state. Our work provides a novel and robust on-chip cavity platform for a wide range of applications, including high-capacity communications, nonlinear optics, atomic clocks, and quantum photonics.

## Abstract (web summary)

The topological edge state cavity (TESC) robustly confines and circulates light through edge states spanning the entire Brillouin zone, enabling simultaneous enhancement of quality factor and free spectral range.

## Graphical abstract

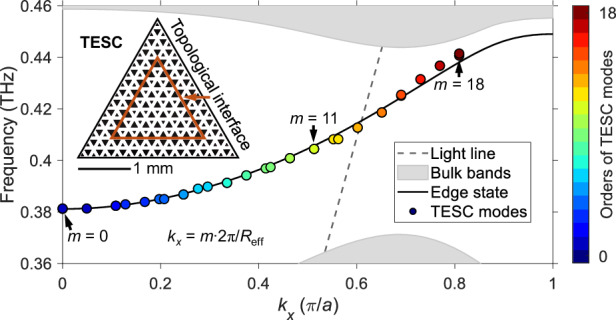

## Introduction

Chip-integrated photonic cavities serve as fundamental building blocks for a variety of applications in photonic integrated circuits, nonlinear photonics, and quantum optics^1–3^. The microscale photonic cavities typically confine and channel the light within a closed geometry through total internal reflection (TIR) or photonic bandgap, enhancing light-matter interactions. At resonant frequencies, light is trapped in the cavity for an extended period, and this temporal enhancement of light-matter interaction is described by the quality factor (*Q*). The free spectral range (FSR) is another important metric, representing the spacing between resonant frequencies. A large FSR is important for achieving stable single-mode lasers and enabling dense frequency-division multiplexing in photonic links^4–6^. The finesse is the ratio of FSR and linewidth of the resonance, which is proportional to the number of round trips made by a photon before it leaves the cavity or is lost through dissipation. A large finesse, requiring both a large FSR and a high *Q*, enables stronger light confinement and longer light-matter interaction times, which are essential for enhancing the performance of various photonic and quantum photonic devices.

**Fig. 1:**
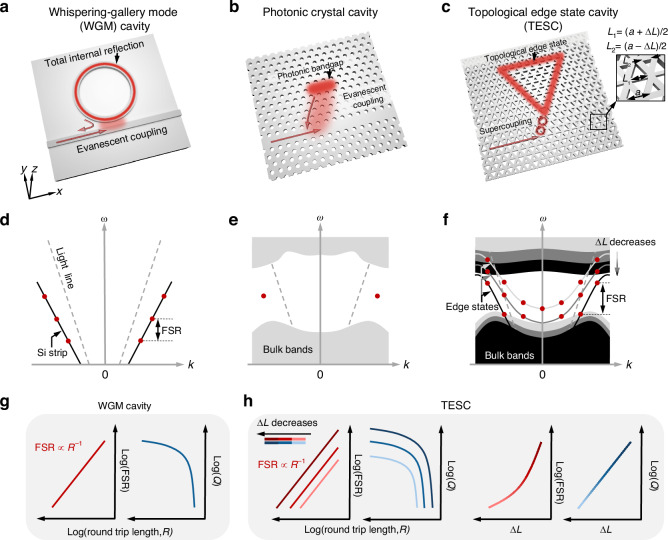
Topological edge state cavity (TESC) and its comparison with conventional whispering-gallery mode (WGM) and photonic crystal (PC) cavities. Schematics of **a** WGM cavity with a microring resonator coupled with a silicon (Si) strip waveguide, **b** PC defect cavity coupled with a line-defect waveguide, **c** TESC coupled with a topological waveguide. Inset of **c** shows the unit cell of valley photonic crystal (VPC) consisting of two equilateral triangular air holes with side lengths *L*_1_ = (*a* + Δ*L*)/2 and *L*_2_ = (*a* ‒ Δ*L*)/2. *a* is the lattice constant. Band diagrams of **d** Si strip, **e** PC, and **f** edge states in VPCs. Waves are confined and guided through total internal reflection (TIR) in WGM cavity, photonic bandgap in PC, and topological edge states in TESC, respectively. When the waves interfere constructively within the cavity, resonant modes (red dots) are formed with discrete momentum (*k*, wavevector) and energy (*ω*, frequency). **g** Evolution of free spectral range (FSR) and quality factor (*Q*) with the round-trip length *R* of WGM cavities. **h** Evolution of FSR and *Q* with *R* and Δ*L* of TESCs. By shifting the edge state from above to below the light line through reducing Δ*L*, as shown in (**f**), the *Q* of TESCs can be enhanced by more than three orders of magnitude, along with a simultaneous improvement in FSR

In general, most of the conventional integrated photonic cavities fall into two categories: whispering-gallery mode (WGM) cavities^2,7–10^ and photonic crystal (PC) cavities^11,12^. In a typical microring WGM cavity (Fig. 1a), the curved silicon strip, with a linear band diagram below the light line (Fig. 1d), confines and guides light through TIR. The finite size of the microring cavity results in quantized wavevectors, leading to discrete WGMs. Enlarging the WGM cavity is an efficient strategy for improving the *Q* by reducing scattering and bending losses. However, this approach also decreases the FSR:

1 $${\rm{FSR}}=\frac{2\pi c}{{Rn}_{g}}$$

where *R* is the round-trip length, *c* is the velocity of light in free space, and *n*_*g*_ is the group index. In contrast, PC cavities confine light in the in-plane direction through a photonic bandgap (Fig. 1e). PC defect cavities with sub-wavelength dimensions are ideal platforms for single-mode applications^11^. However, both high *Q* resonances and multi-mode operation require careful design of the cavity, as those are extremely sensitive to the fabrication imperfections and disorders.

The emerging field of topological valley photonics offers a novel paradigm for light confinement with topological protection by harnessing the valley degree of freedom^13–16^. The topological edge states robustly guide on-chip light smoothly traveling through sharp bends with negligible bending loss. By bending the topological interface into a closed loop (Fig. 1c), the topological cavity not only confines light at the domain wall through the topological bandgap but also guides it to robustly circulate along the cavity, establishing resonances through the topological edge states. Several works have demonstrated topological cavities in triangular^14,17,18^ and parallelogram shapes^19,20^. However, the underlying mechanism of the topological cavity modes is still not well understood, which is essential for the effective design of integrated high-*Q* cavities with a large FSR.

Here, we reveal the physical mechanism behind the emergence of topological cavity modes, which we term as topological edge state cavity (TESC) modes, demonstrating that they originate from the topological edge states at discrete wavenumbers imposed by the finite cavity size (Fig. 1f). Similar to WGM cavities, the TESC easily achieves high *Q* and control over FSR by simply enlarging the cavity size (Fig. 1h). Notably, by shifting the topological edge states from above to below the light line through modifying the spatial inversion symmetry of valley photonic crystals (VPCs), we show that the intrinsic *Q* (*Q*_in_) of TESC modes is dramatically enhanced by three orders of magnitude from 67 to 219,520, while FSR increases from 5.1 to 7.1 GHz simultaneously. We experimentally demonstrate the on-chip TESC and realize a 141-fold enhancement in the measured *Q*_in_, increasing from 124 for the 0th-order leaky TESC mode to 17,521 for the guided TESC mode.

## Results

### Design of TESCs

**Fig. 2:**
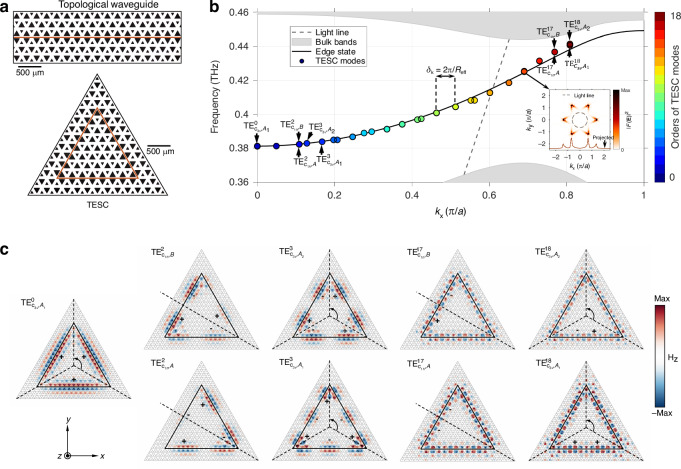
Dispersion relations and symmetries of TESC modes. **a** Optical images of topological waveguide with a straight zigzag interface (brown line) and TESC with the zigzag interface forming a triangular closed path. The lattice constant *a* = 220 μm and Δ*L* = 0.3*a*. The side length of the TESC is *L* = 8*a*. **b** Calculated band diagram of TESC modes, presenting as topological edge states with discrete wavevectors. The wavevector of TESC modes is determined at the *k*_*x*_ with the maximum intensity of the projected field in the momentum space, which is analyzed through spatial Fourier transformation of the electric field of eigen TESC modes (inset of **b**). Here Δ*L* = 0.7*a* and *L* = 16*a* are designed to show the 0th-order TESC mode at Γ point. *δ*_*k*_ = 2π/*R*_eff_ represents the wavevector spacing between TESC modes, which is governed by the effective round-trip length of the cavity, *R*_eff_. **c** Simulated magnetic field *H*_*z*_ distributions of the eigen TESC modes. The TESC modes are noted as ${\text{TE}}_{P,I}^{m}$, where TE denotes transverse electric modes, *m* = 0, 1, 2, 3, … represents the order of modes, *P* = *C*_3*v*_, *C*_1*h*_ corresponds to the point group, and *I* = *A*, *B* or *A*_1_, *A*_2_ represent the irreducible representations of the point groups *C*_1*h*_ and *C*_3*v*_, respectively. The dashed black lines indicate the reference lines where the mirror operation is applied

We start with a topological waveguide design featuring a straight zigzag interface (Fig. 2a). The silicon VPC slab has a lattice constant *a* = 220 μm and a thickness of 190 μm. The unit cell consists of two equilateral triangular air holes with side lengths *L*_1_ = (*a* + Δ*L*)/2 and *L*_2_ = (*a* ‒ Δ*L*)/2 (inset of Fig. 1c). When the spatial inversion symmetry of VPC is broken with Δ*L* = 0.7*a*, the topological zigzag interface supports a single edge state that spans nearly the entire Brillouin zone within the bandgap (Fig. 2b and Supplementary Information section 1). To construct a closed-path cavity, we bend the zigzag interface into a triangular shape, ensuring a single type of interface in TESC. Since the size of the cavity is finite, the wavevector of TESC modes is quantized: *k*_res_ = *m*∙2*π*/*R*_eff_, where *m* represents the order of TESC modes (0, 1, 2, 3…), and *R*_eff_ is the effective round-trip length.

As a specific example, we consider a TESC with a side length of *L* = 16*a*. Two-dimensional (2D) spatial Fourier transformation of the electric field **E** distribution for the eigen TESC modes is conducted. The wavevector of TESC modes is determined at the *k*_*x*_ with the maximum intensity of the projected field ${\left|{\mathcal{F}}\left({\bf{E}}\right)\right|}^{2}$ in the momentum space (inset of Fig. 2b). The dispersion relations of TESC modes indicate that they are topological edge states with discrete wavevectors. In other words, the frequency of the *m*th-order TESC modes is determined by the frequency of the edge state at *k*_*x*_ = *m* 2π*/R*_eff_. Ideally, the TESC modes are equally separated in the momentum space with a spacing of *δ*_*k*_ = 2π/*R*_eff_. However, the lower-order TESC modes, with longer effective wavelengths, take a larger “shortcut” at the corners of the cavity through the Poynting vector flow vortices (Supplementary Information section 2), resulting in a smaller *R*_eff_ and a larger *δ*_*k*_. The largest FSR is obtained near the K and K’ valleys where the dispersion curve of the edge state has the steepest local slope, resulting in the minimum *n*_*g*_ (Supplementary Information section 3). Moreover, the TESC modes span nearly the entire Brillouin zone of the topological interface, residing both above and below the light line. This enables their excitation and coupling in both free-space and on-chip configurations, thereby enhancing the functionality of TESCs.

According to the in-plane symmetries, the TESC modes are noted as:

2 $${\text{TE}}_{P,I}^{m}$$

where TE denotes transverse electric modes, *P* = *C*_3*v*_, *C*_1*h*_ corresponds to the point group, and *I* = *A*, *B* or *A*_1_, *A*_2_ represents the irreducible representations of point groups *C*_1*h*_ and *C*_3*v*_, respectively (Supplementary Information section 4). For ${\text{TE}}_{C3v,A1}^{0}$ mode at Γ point, the phase remains constant along the sides of the cavity (Fig. 2c), resulting in *C*_3_ symmetry and single-mode behavior. When *m* = 3*N* (*N* = 1, 2, 3…), the paired TESC modes with broken degeneracy also have *C*_3_ symmetry and exhibit either even symmetry (denoted as representation *A*_1_ in point group *C*_3*v*_) or odd symmetry (representation *A*_2_) under mirror operation. When *m* = 1 + 3*N* or 2 + 3*N*, the number of wavelengths within the cavity is not an integer multiple of three, which is the number of cavity’s sides. As a result, the paired TESC modes only exhibit either even (denoted as representation *A* in point group *C*_1*h*_) or odd (representation *B*) mirror symmetry and present degenerate features. We should note that the paired TESC modes arise from the mirror symmetries, rather than the clockwise and counterclockwise properties typically seen in degenerate WGMs.

### *Q* and FSR scaling of TESCs

**Fig. 3:**
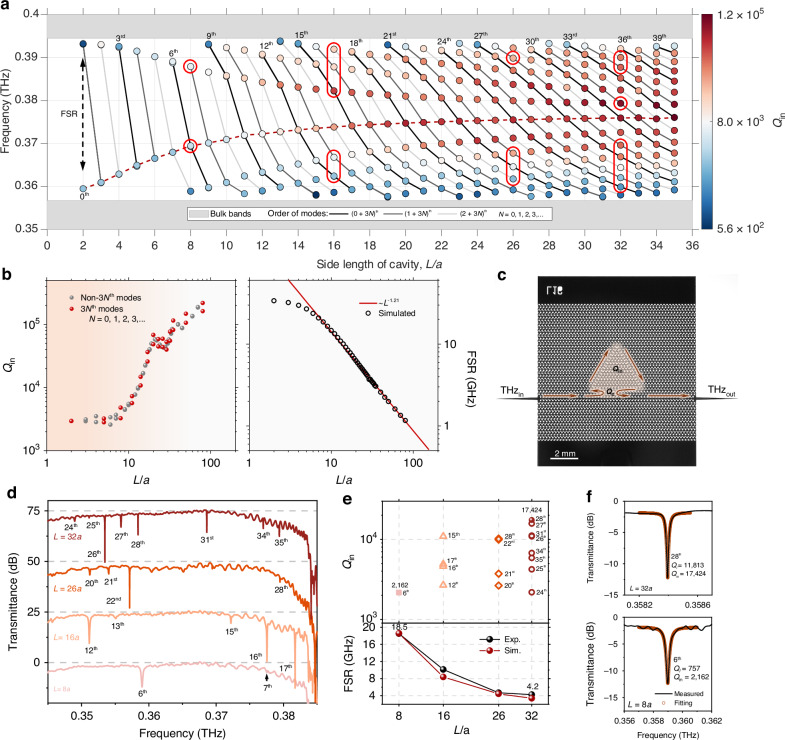
*Q* and FSR scaling of TESCs. **a** Evolution of TESC modes with the side length of the cavity (*L*). The TESC modes of the same order are connected by a solid line. As *L* increases, FSR decreases while the intrinsic quality factor (*Q*_in_) rises, which is indicated by color variation. **b** Scaling of FSR and *Q*_in_ of the TESC mode having the largest FSR, which is indicated by a red dashed line in (**a**). **c** Optical image of a TESC chip with *L* = 16*a*. The TESC is coupled and excited by a topological waveguide, separated by four unit cells. The coupling quality factor (*Q*_*c*_) accounts for the coupling loss. **d** Measured transmittance spectra of TESC chips with *L* = 8*a*, 16*a*, 26*a*, and 32*a*. The measured TESC modes are marked by red circles in (**a**). They shift to the lower frequency by ~10.3 GHz compared to the simulated eigen results in (**a**) due to the higher refractive index of silicon wafer relative to the value used in the simulation (3.32). **e** Extracted *Q*_in_ and maximum FSR of TESC chips with different *L*. **f** Measured and fitted transmittance spectra of TESC modes. Δ*L* is fixed at 0.3*a*

As with WGM cavities, scaling the size of TESCs is a straightforward and effective method for engineering the *Q* and FSR of TESC modes. When TESC becomes larger, the smaller momentum spacing *δ*_*k*_ results in the emergence of more TESC modes within the topological bandgap, as shown in Fig. 3a for Δ*L* = 0.3*a*. We track the mode with the largest FSR and analyze the scaling of its *Q*_in_ and FSR with the size of the cavity (*L*). Light circulating within the TESC experiences five main types of losses without considering material loss: radiation loss (*l*_*r*_) due to mode energy leakage into the light cone, propagation loss (*l*_*p*_), bending loss (*l*_*b*_) at the three corners, scattering loss (*l*_*i*_) from side-to-side interference, and bulk-scattering loss (*l*_*s*_). When *L* increases from 2*a* to 20*a*, the TESC mode shifts to higher frequency where the edge state moves farther from the light line and has a smaller effective wavelength (increased *k*_res_), resulting in reduced *l*_*r*_ and *l*_*b*_. Additionally, a large TESC reduces *l*_*i*_, causing a rapid increase in *Q*_in_ (Fig. 3b). As *L* further increases, *l*_*r*_ and *l*_*b*_ stabilize, but the continuously decreasing *l*_*i*_ causes *Q*_in_ to keep rising, albeit more gradually. Interestingly, the highest *Q*_in_ of a TESC with a fixed size (for example, *L* = 35*a*) is obtained near the center of the topological bandgap, where *l*_*s*_ is minimized and *l*_*r*_ and *l*_*b*_ remain relatively low. On the other hand, a larger TESC has an increased effective round-trip length (*R*_eff_), resulting in a smaller FSR, as described by the scaling law in Eq. (1). The deviation of FSR_TESC_ ∝ *L*^−1.21^ from *L*^−1^ is attributed to *R*_eff_ < 3*L*, which is induced by the “shortcut” of the Poynting vector flow at the corners of the cavity (Supplementary Information section 2).

We fabricate the TESC chips on a high-resistivity silicon wafer (see Methods for the details of fabrication). Figure 3c shows the optical image of a silicon TESC with *L* = 16*a* and Δ*L* = 0.3*a*. It is coupled and excited by a topological waveguide featuring a different type of zigzag interface (Supplementary Information section 5) through Poynting vector flow vortices-enabled supercoupling^21^. The coupling quality factor (*Q*_*c*_) accounts for the coupling loss. When *L* = 8*a* and the coupling distance is seven unit cells, the 6th-order TESC mode with a strong resonance dip is observed at 0.359 THz. Compared to the eigen results shown in Fig. 3a, the measured TESC mode shifts to the lower frequency by ~10.3 GHz, due to the higher refractive index of the silicon wafer relative to the value used in the simulation (3.32). As *L* increases to 32*a* with a coupling distance of six unit cells, eight TESC modes are observed, ranging from the 24th to the 35th order. Some TESC modes are not visible in the transmittance spectra and do not feature a resonant dip due to their strongly overcoupled state. We extract the loaded quality factor (*Q*_*l*_) by fitting the transmittance spectra to a Fano function (Fig. 3f) and subsequently obtain *Q*_in_ from coupled mode theory (Supplementary Information section 6). Figure 3e shows that the measured *Q*_in_ increases from 2162 to 17,424 as *L* increases from 8*a* to 32*a*. The maximum FSR of each TESC chip is determined from the group delay spectra, where the TESC modes, although hidden in the transmittance spectra, still exhibit resonant features (Supplementary Information section 7). The measured FSR decreases by 4.4 times, from 18.5 to 4.2 GHz.

### Simultaneously enhancing *Q* and FSR

**Fig. 4:**
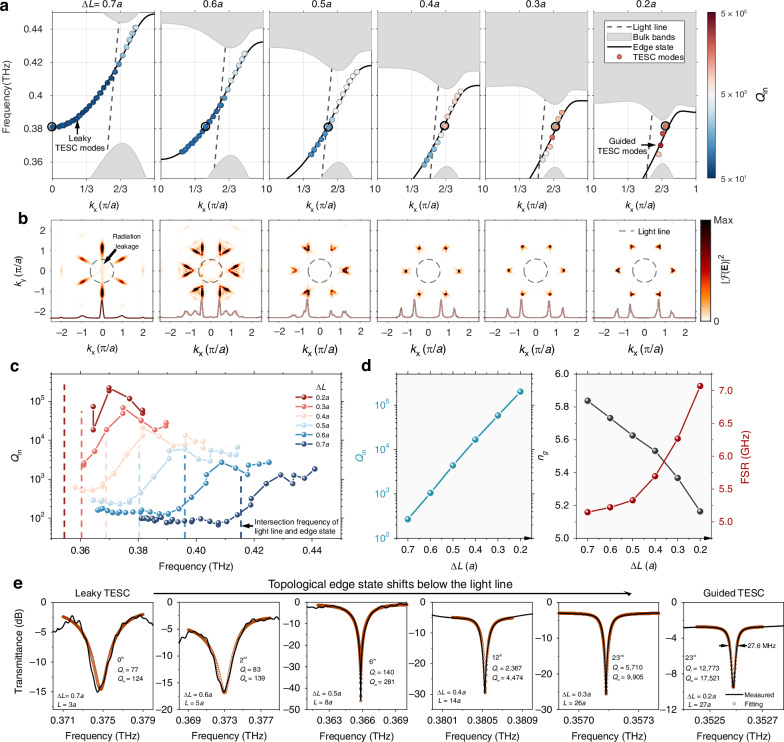
Simultaneously enhancing both *Q*_in_ and FSR of TESC through edge state engineering. **a** Evolution of the edge state and TESC modes with Δ*L*. As Δ*L* decreases from 0.7*a* to 0.2*a*, the edge state initially above the light line is shifted below it, leading to the leaky TESC modes transitioning into guided TESC modes, with *Q*_in_ increasing from 67 to 219,520. Here *L* is fixed at 20*a*. **b** Fourier transformation of the electric field of eigen TESC modes near 0.38 THz for different Δ*L*, along with the projected distribution of ${\left|{\mathcal{F}}\left({\bf{E}}\right)\right|}^{2}$ along the wavevector *k*_*x*_. The TESC modes are marked with circles in **a**. As Δ*L* decreases from 0.7*a* to 0.2*a*, the radiation leakage inside the light cone disappears. **c** Simulated *Q*_in_ of TESC modes for different Δ*L* with a fixed *L* = 20*a*. As the TESC modes shift above the intersection frequency of light line and edge state, *Q*_in_ increases dramatically. **d** Simulated *Q*_in_ and FSR of TESC modes and maximum *n*_*g*_ of edge state for different Δ*L*. Both *Q*_in_ and FSR are enhanced by reducing Δ*L*. **e** Measured and fitted transmittance spectra of TESC modes. As Δ*L* decreases from 0.7*a* to 0.2*a*, *Q*_in_ increases from 124 to 17,521. The coupling distance between the TESC and topological waveguide is 2, 2, 3, 4, 6 and 12 unit cells for Δ*L* ranging from 0.7*a* to 0.2*a*

Since the TESC modes inherit the dispersion relation properties of topological edge state, engineering edge state offers a novel approach to controlling both the *Q* and FSR of TESC modes. Here, we demonstrate that by controlling the spatial inversion symmetry of VPCs (Δ*L*) to modify the radiation leakage and group index of topological edge state, both *Q* and FSR of TESC modes can be enhanced simultaneously. Figure 4a shows that as Δ*L* decreases from 0.7*a* to 0.2*a*, the edge state initially residing above the light line is shifted below it, effectively suppressing radiation leakage. We calculate the eigenmodes of a TESC with *L* = 20*a* for different Δ*L*. The TESC modes located above the light line, where their projected momentum *k*_*x*_ is smaller than the free-space wavenumber *k*_0_, exhibit strong radiation and are termed leaky TESC modes. In contrast, those below the light line, where *k*_*x*_ > *k*_0_ and the TIR condition is satisfied, have strong out-of-plane field confinement and are referred to as guided TESC modes. As Δ*L* decreases, the field ${\left|{\mathcal{F}}\left({\bf{E}}\right)\right|}^{2}$ of TESC modes gradually shifts out of the light cone in the momentum space (Fig. 4b), clearly demonstrating the suppression of radiation leakage. As a result, *Q*_in_ increases by more than three orders of magnitude, from 67 to 219,520. Interestingly, the intersection frequency of light line and edge state marks the transition point, below which *Q*_in_ remains nearly constant, and then rapidly increases after crossing it (Fig. 4c). Additionally, as the edge state is shifted downward, its slope becomes steeper. The group velocity ${v}_{g}=d\omega /{dk}$ increases and ${n}_{g}=c/{v}_{g}$ decreases, leading to an enhancement of FSR as described in Eq. (1). Figure 4d shows that the maximum FSR increases from 5.1 to 7.1 GHz and *Q*_in_ rises from 267 to 201,715 as Δ*L* decreases from 0.7*a* to 0.2*a*. To experimentally demonstrate the edge state engineering-enabled control over *Q* of TESC modes, we fabricated TESC chips with different Δ*L*. *L* and coupling distance are optimized for each Δ*L* to ensure clear and strong TESC resonances while avoiding the merging of resonances in the transmittance spectrum, particularly for low-*Q* TESC modes with a large Δ*L*. Figure 4e shows that as Δ*L* decreases from 0.7*a* to 0.2*a*, the 0th-order leaky TESC mode transitions into a guided TESC mode, with the measured *Q*_*l*_ (*Q*_in_) increasing by 166 (141) times from 77 to 12,773 (from 124 to 17,521).

## Discussion

In conclusion, we have experimentally demonstrated a novel on-chip photonic cavity platform, TESC, capable of simultaneously enhancing both *Q* and FSR. Utilizing spatial Fourier transformation, we revealed that the TESC modes originate from the topological edge state at discrete wavenumbers in a finite-sized, closed-path topological interface. The TESC confines light through a topological photonic bandgap and robustly guides it to circulate around the cavity via the local Poynting vector flow vortices. The TESC retains the ease of achieving high *Q* and controlling the FSR of WGM cavities by simply increasing the cavity size. Importantly, by controlling the spatial inversion symmetry of VPCs to modify the radiation leakage and group index of topological edge state, we demonstrated that the *Q*_in_ of TESC modes is dramatically enhanced by three orders of magnitude from 67 to 219,520, while the FSR increases from 5.1 to 7.1 GHz simultaneously. By driving the edge state across the light line, from above to below, we experimentally achieved a 141-fold enhancement in *Q*_in_, increasing from 124 for the leaky TESC mode to 17,521 for the guided TESC mode. It should be noted that while here we exploit only the spatial inversion symmetry of VPCs to tailor the topological edge state, interfacial engineering of topological waveguides could potentially provide even greater flexibility in shaping the edge state^22,23^, and thus offer larger control over the *Q* and FSR of TESC modes. For example, constructing a TESC using a bearded interface with a slow-light edge state could further enhance light-matter interactions. The simultaneous enhancement of *Q* and FSR leads to a significant increase in finesse, making TESCs highly promising for applications in frequency filtering and spectroscopy requiring high spectral resolution, as well as in nonlinear optics and quantum electrodynamics, where strong light-matter interactions are essential. Our TESC provides a novel and robust on-chip cavity platform, which is promising to unlock a plethora of new applications in photonic integrated circuits, nonlinear photonics, and quantum optics.

## Materials and methods

### TESC chip fabrication

The TESC chip was fabricated using a 6-inch 500 μm thick high-resistivity ( > 20 kΩ ·cm) silicon wafer. First, a 2.5 μm thick silicon dioxide (SiO_2_) layer was deposited as an etching protective mask. Then, the TESC design was patterned on the SiO_2_ layer using a conventional UV photolithography process followed by reactive ion etching (RIE). The remaining pattern of SiO_2_ layer serves as a protective mask and the silicon wafer was etched to a depth of 300 μm using deep reactive ion etching (DRIE, Oxford Estrelas), ensuring straight sidewalls for more than 190 μm. Finally, the etched silicon wafer was back grinded to a thickness of 190 μm.

### Numerical simulation

The band structure of the VPC unit cell and edge states of the topological interface were calculated using the eigenvalue solver of the commercial software COMSOL Multiphysics. The dielectric constants of air and silicon were set to 1 and 11, respectively. Floquet boundary conditions were applied in both *x*- and *y*-directions in the VPC unit cell, and in only *x*-direction at the topological interface. The commercial software CST Studio Suite was used to calculate the *Q*_in_ of TESC using the eigenmode solver and simulate the transmittance of the TESC-waveguide coupled system using the time domain solver. The VPC bulk area surrounding the TESC was optimized to be sufficiently large to ensure that the energy of TESC modes is well confined in the in-plane direction without leaking from the sides of the cavity, while also avoiding excessive computational memory requirements. Open (add space) conditions were applied in all directions. The materials were defined as the same as in the eigenmode calculations using COMSOL Multiphysics.

### On-chip TESC transmittance characterization setup

The transmittance of the TESC chips was characterized using the vector network analyzer (VNA) setup. A microwave signal ranging from 10 MHz to 26.5 GHz was first generated by the Keysight N5222B network analyzer. It was then upconverted to 330–500 GHz using WM-570 VNAX frequency extension modules. The output signal passing through the TESC chips was down-converted by another WM-570 VNAX frequency extension module and finally analyzed by the Keysight network analyzer. The system was calibrated using a SOLT (Short-Open-Load-Through) waveguide calibration procedure adhering to WR-2.2 waveguide standards.

## Supplementary information

Supplementary Information

## Data Availability

All the data in this study are openly available in the NTU research data repository DR-NTU at 10.21979/N9/XBMXRT. Additional information related to this paper should be addressed to Ranjan Singh (rsingh3@nd.edu).
