# Supplementary material for: On-chip topological edge state cavities
Source: Light Sci Appl. 2025 Sep 18;14:330. doi: 10.1038/s41377-025-02017-3 (PMC12446465; doi:10.1038/s41377-025-02017-3)
Supplement: Supplementary file 1 — Supplementary Information [file 41377_2025_2017_MOESM1_ESM.pdf]

Supplementary Information for

On-chip topological edge state cavities

Wenhao Wang[1,2], Zhonglei Shen[1,2], Yi Ji Tan[1,2], Kaiji Chen[1,2] and Ranjan Singh[1,2,3]*

[1]*Division of Physics and Applied Physics, School of Physical and Mathematical Sciences, Nanyang Technological University; Singapore 637371, Singapore.*
[2]*Centre for Disruptive Photonic Technologies, The Photonics Institute, Nanyang Technological University; Singapore 637371, Singapore.*
[3]*Department of Electrical Engineering, University of Notre Dame, Notre Dame, IN, USA.*

*Email: rsingh3@nd.edu

This file includes:

S1. Eigenvalue analysis of valley photonic crystals (VPCs)

S2. Shortcut of Poynting vector flow at the corners of TESC

S3. Maximum FSR near the K (K') valleys

S4. Symmetries of TESC modes

S5. Coupling and excitation of TESC

S6. Extraction of Q_{in} and Q_c through coupled mode theory

S7. Determining FSR from the group delay spectra

S8. Comparison of TESCs with existing topological cavities

S9. Robustness of TESCs against structural disorders

S10. Effect of material loss on the Q factor of TESC

S11. Comparison of TESC with WGM and conventional photonic crystal (PC) cavities

Additional references in Supplementary Information

S1. Eigenvalue analysis of valley photonic crystals (VPCs).

The rhombus-shaped VPC unit cell consists of two equilateral air holes with side lengths L_1 and L_2 (Fig. S1a). When $\Delta L = L_1 - L_2 = 0$, the VPC unit cell has inversion symmetry and C_6 symmetry, leading to a pair of degenerate Dirac points at K and K' valleys (Fig. S1b). When the inversion symmetry is broken, for instance with $\Delta L = \pm 0.7a$, the VPC unit cell retains only C_3 symmetry. The degeneracy of the Dirac points is lifted, and a topological bandgap emerges. At K valley, the VPC unit cell with $\Delta L = 0.7a$ exhibits a vortex distribution of the magnetic field H_z and a clockwise Poynting power flow vortex at the center of the silicon region (Fig. S1c). In contrast, the VPC unit cell with $\Delta L = -0.7a$ exhibits an opposite distribution of the magnetic field H_z and a counterclockwise Poynting power flow vortex. This indicates that the two types of VPC unit cells support different circularly polarized eigenstates. Moreover, for a given type of VPC, the eigenstate exhibits opposite circular polarization at K and K' valleys, showing valley-dependent features.

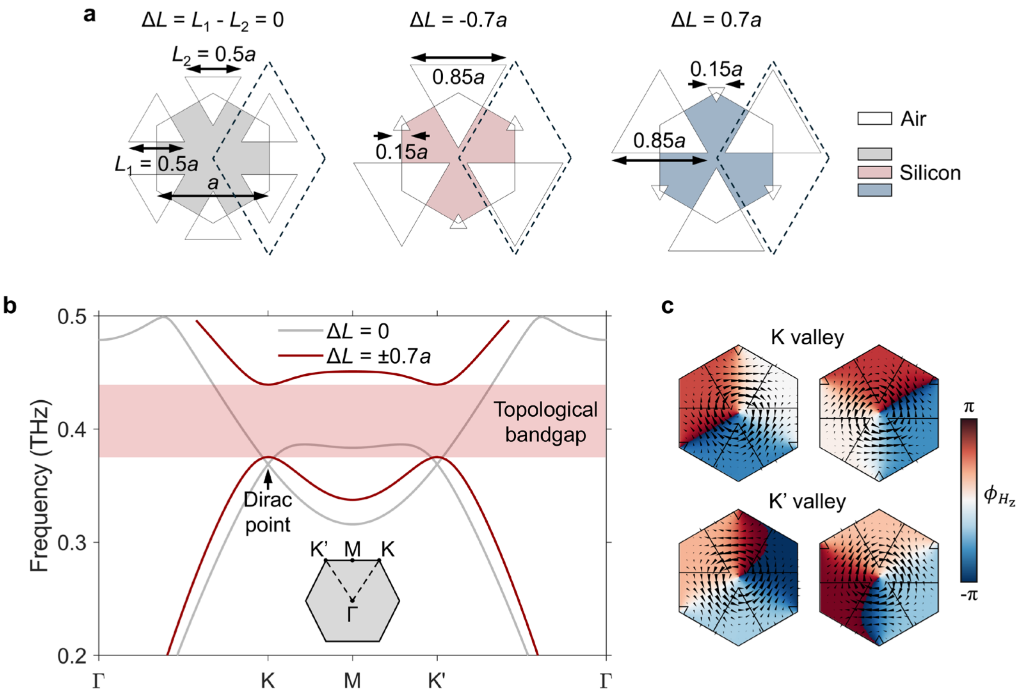

Fig. S1 | Topological valley photonic crystals (VPCs) and band structures. a, Unit cells of VPC for $\Delta L = 0$, $-0.7a$, and $0.7a$. The rhombus-shaped VPC unit cell, plotted with dashed black lines, consists of two equilateral air holes with side lengths L_1 and L_2. The lattice constant $a = 220$ μm. **b**, Calculated band structures of VPC for $\Delta L = 0$, $-0.7a$, and $0.7a$. Inset shows the first Brillouin zone of VPC. **c**, Calculated phase distributions of the magnetic field H_z of the lower band for VPC unit cell with $\Delta L = 0.7a$ and $-0.7a$. The black arrows denote the Poynting vector flow.

The topological phase of VPC is characterized by the valley Chern number, obtained by integrating Berry curvatures around the valleys. Fig. S2 shows the distribution of Berry curvature for VPCs with different ΔL. As $|\Delta L|$ increases from $0.1a$ to $0.7a$, the Berry curvature becomes delocalized from the K and K' valleys. The VPC with $\Delta L = 0.7a$ exhibits a Berry curvature profile with opposite signs compared to the VPC with $\Delta L = -0.7a$, indicating that they have opposite nonzero valley Chern numbers and opposite topological phases. By stacking VPCs with $\Delta L = \pm 0.7a$ to form a zigzag interface (Fig. 2a), a single valley-polarized topological edge state emerges due to bulk-boundary correspondence (Fig. 2b).

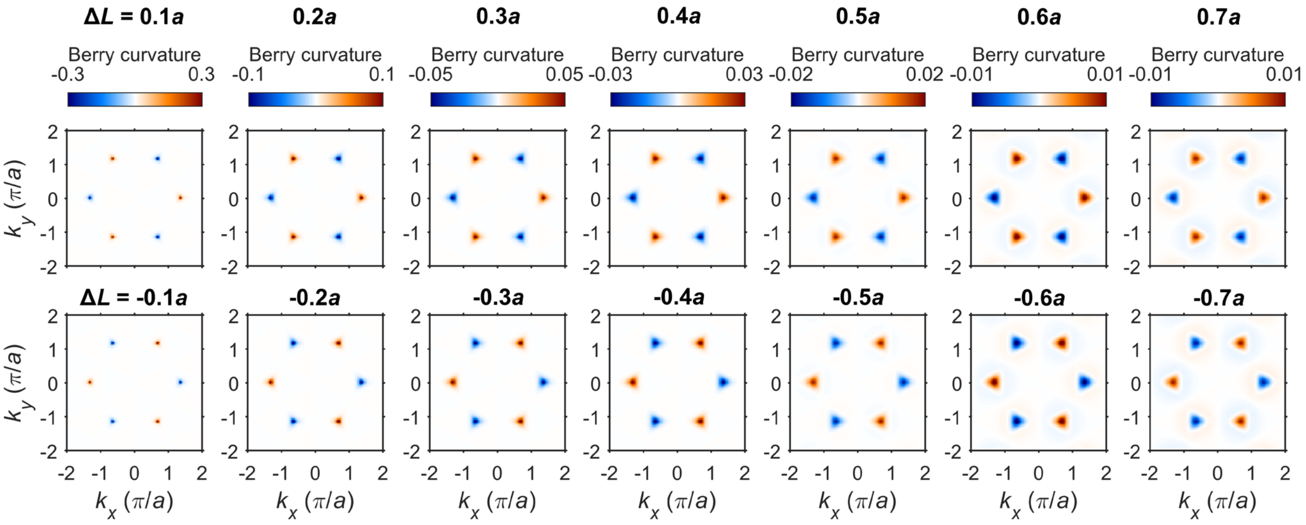

Fig. S2 | Distribution of Berry curvature for VPCs. Calculated distributions of Berry curvature for VPCs with ΔL varying from $\pm 0.1a$ to $\pm 0.7a$.

S2. Shortcut of Poynting vector flow at the corners of TESC

We use 2D simulation in COMSOL Multiphysics with a frequency domain solver to calculate how light is guided and channeled within the TESC. The triangular TESC with $\Delta L = 0.8a$ has a side length of $L = 16a$ and a bulk area of $15a$ surrounding the cavity. Scattering boundary conditions are applied to the triangular boundaries of the structure. We use an electric point dipole source, $E = E_x + iE_y$ (with the electric field rotating counterclockwise), placed near the TESC to excite the 15th-order TESC mode. Fig. S3 shows the simulated Poynting vector flow near the bottom left corner of the TESC. The Poynting vector flow vortices guide the light smoothly traveling through the bend, taking a shortcut before reaching the corner. The intensity distribution of the Poynting vector flow clearly

demonstrates that the effective round-trip length of the TESC mode is shorter than the physical circumference of the cavity: $R_{\mathrm{eff}} < 3L$.

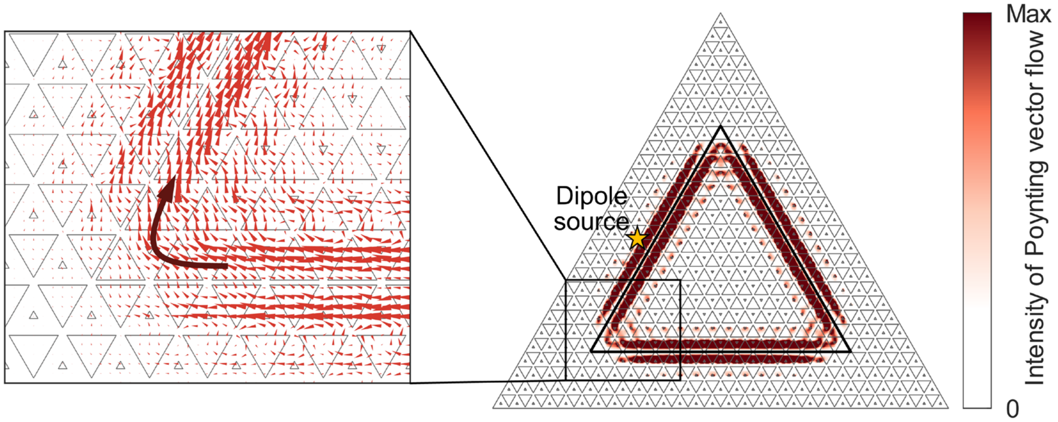

Fig. S3 | Shortcut routing of Poynting vector flow at the corners of TESC. Simulated Poynting vector flow (left panel) and its intensity distribution (right panel) of the 15th-order TESC mode which is excited by an electric point dipole source $E = E_x + iE_y$.

Fig. S4 shows the extracted R_{eff} of TESC modes for $\Delta L = 0.7a$ and $L = 16a$, calculated using $R_{\mathrm{eff}} = m \cdot 2\pi/k_m$, where m represents the order of TESC modes and k_m is obtained through spatial Fourier transformation of the electric field of eigen TESC modes. For lower-order TESC modes, the longer effective wavelength results in a larger shortcut at the corners of TESC, leading to a smaller R_{eff}. As the mode order increases, the effective wavelength of TESC mode decreases and the smaller shortcut yields a larger R_{eff}. For the highest-order 18th TESC mode, R_{eff} approaches $45a$, indicating that the on-chip wave follows a path that is one unit cell shorter than the geometric length at each corner.

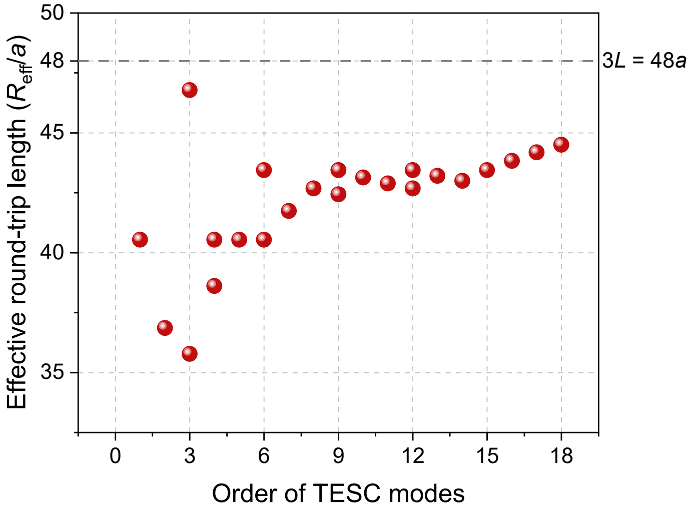

Fig. S4 | Effective round-trip length, R_{eff}, of TESC modes for ΔL = 0.7a and L = 16a. R_{eff} is extracted using $R_{eff} = m \cdot 2\pi/k_m$, where m represents the order of TESC modes and the momentum k_m is obtained through spatial Fourier transformation of the electric field of eigen TESC modes.

S3. Maximum FSR near the K (K') valleys

Fig. S5 shows the n_g of the edge state for the topological zigzag interface with $\Delta L = 0.3a$. The minimum n_g of 5.37 is obtained at 0.377 THz near the K (K') valleys. As shown in equation (1), a minimum n_g will result in the maximum FSR for a TESC with a fixed effective round-trip length R_{eff}. To investigate this, we calculate the eigenmodes of a TESC with a large side length of $L = 35a$ to obtain more TESC modes. The calculated FSR initially increases and then decreases as the frequency rises. The maximum FSR of 3.04 GHz is achieved at 0.379 THz, near the valleys.

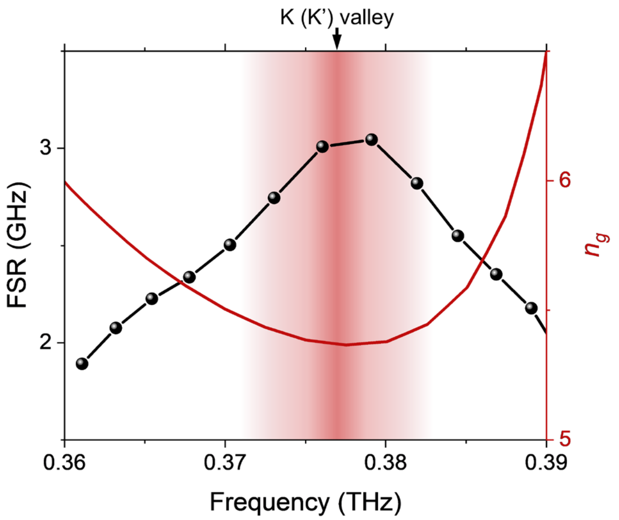

Fig. S5 | Maximum FSR near the K (K') valleys. Calculated FSR of eigen TESC modes for a TESC with ΔL = 0.3a and L = 35a, and n_g of the topological edge state for the zigzag interface with ΔL = 0.3a.

S4. Symmetries of TESC modes

The TESC modes with an order of m = 3N (N = 0, 1, 2, 3…) exhibit both C_3 symmetry and mirror symmetry, corresponding to the point group symmetry C_{3v}. Specifically, modes with the A_1 (A_2) irreducible representation show a symmetric (antisymmetric) profile under the mirror symmetry operation σ. For example, $\mathrm{TE}^{0}_{C_{3v},A_1}$ exhibits symmetry, while $\mathrm{TE}^{3}_{C_{3v},A_2}$ is antisymmetric. Table S1 shows the irreducible representations and their character tables for the C_{3v} point group[1]. However, the TESC modes with an order of m = 1+3N or m = 2+3N (N = 0, 1, 2, 3…) possess only mirror symmetry, corresponding to the point group symmetry C_{1h}. The modes with A (or B) irreducible representation exhibit symmetric (or antisymmetric) profiles after applying mirror symmetry operation σ. The irreducible representations and their character tables for the C_{1h} point group are shown in Table S2.

Table S1. The character table for the C_{3v} point group

C_{3v}	E	C_3	σ
A_1	1[a]	1	1
A_2	1	1	-1[a]

[a]The number 1 and -1 indicate a symmetric and antisymmetric profile after applying symmetry operations (E, C_3, σ), respectively.

Table S2. The character table for the C_{1h} point group

C_{1h}	E	σ
A	1	1
B	1	-1

S5. Coupling and excitation of TESC

The TESC is coupled and excited through the topological waveguide that features a different type of zigzag interface. As shown in Fig. S6a, the topological waveguide, bent into a triangular closed path to form TESC, consists of VPCs with $\Delta L = 0.3a$ on the upper side and $\Delta L = -0.3a$ on the lower side of the topological zigzag interface. The edge state supported by the topological zigzag interface is shown in Fig. S6b. To efficiently excite the TESC, the cavity is coupled with and excited by a different type of topological waveguide in a side-by-side configuration. The excitation waveguide has the VPCs with $\Delta L = -0.3a$ on the upper side and $\Delta L = 0.3a$ on the lower side of the topological zigzag interface. Fig. S6b shows that the two types of edge states have opposite group velocities at the same valley. When the THz wave is channeled into the topological chip through the taper coupler, it propagates forward, carrying momentum near the K' valley. In the coupling region, the THz wave flows into the cavity through the valley vortices-enabled supercoupling. Since the THz wave has K' valley-locked momentum, the excited edge state in the TESC guides the THz wave to propagate backward and circulate clockwise. It should be noted that for the TESCs with different ΔL, the excitation waveguide is constructed with the same ΔL, but with the VPCs on each side swapped.

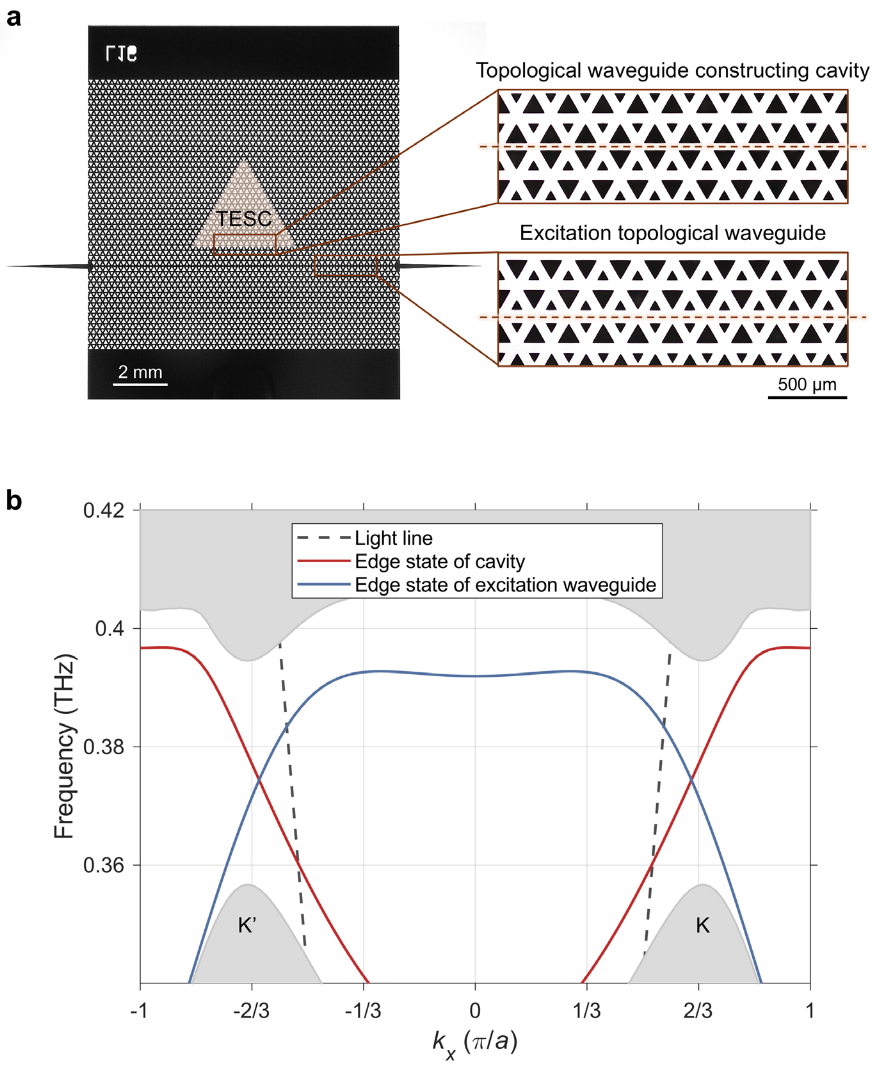

Fig. S6 | Excitation of TESC. **a**, Optical image of a TESC chip with L = 16a. The TESC is constructed by the topological waveguide consisting of VPCs with ΔL = 0.3a on the upper side and ΔL = -0.3a on the lower side of the zigzag interface. The excitation waveguide has the same ΔL, but the VPCs on each side are swapped. **b**, Calculated band diagram of topological edge states.

S6. Extraction of Q_{in} and Q_c through coupled mode theory

We extract the Q_l of each TESC resonance by fitting the transmittance spectra $T(f)$ to a Fano function:

$$T(f) = T_0 + I\frac{(W+q)^2}{(1+q)^2(1+W^2)}, \quad W = \frac{f - f_{TESC}}{\gamma/2} \tag{S1}$$

where f_{TESC}, γ, and I are frequency, linewidth, and normalized intensity of TESC modes, T_0 is the baseline shift of the whole spectrum, and q is the asymmetry parameter. The Q_l is determined by $Q_l = f_{TESC}/\gamma$. According to the coupled mode theory, the transmittance at the TESC resonant frequency is

$$T_{\mathrm{TESC}} = \left| \frac{Q_c - Q_{\mathrm{in}}}{Q_c + Q_{\mathrm{in}}} \right|^2 \quad \text{(S2)}$$

Since $1/Q_l = 1/Q_c + 1/Q_{\mathrm{in}}$, we can easily derive $Q_c = 2Q_l/(1 + \sqrt{T_{\mathrm{TESC}}})$ and $Q_{\mathrm{in}} = 2Q_l/(1 - \sqrt{T_{\mathrm{TESC}}})$ when the TESC is overcoupled, and $Q_c = 2Q_l/(1 - \sqrt{T_{\mathrm{TESC}}})$ and $Q_{\mathrm{in}} = 2Q_l/(1 + \sqrt{T_{\mathrm{TESC}}})$ when the TESC is undercoupled.

S7. Determining FSR from the group delay spectra

The upper panel of Fig. S7 shows the transmittance of the TESC chip with $\Delta L = 0.3a$ and $L = 32a$. Since the 29th and 30th-order TESC modes are in a strongly overcoupled state, they are not visible in the transmittance spectra and do not feature resonant dips. The phase response $\phi(\omega)$ is generally more sensitive to resonance features than the amplitude response when sweeping the frequency across a resonance. Since the group delay is defined as $\tau_g(\omega) = -d\phi(\omega)/d\omega$, it is even more sensitive to subtle phase variations. As a result, the 29th and 30th-order TESC modes, which are although not visible in the transmittance spectrum, still produce weak but clear resonant peaks in the group delay spectrum (lower panel of Fig. S7). The maximum FSR of 4.2 GHz is obtained between the 29th and 30th-order TESC modes.

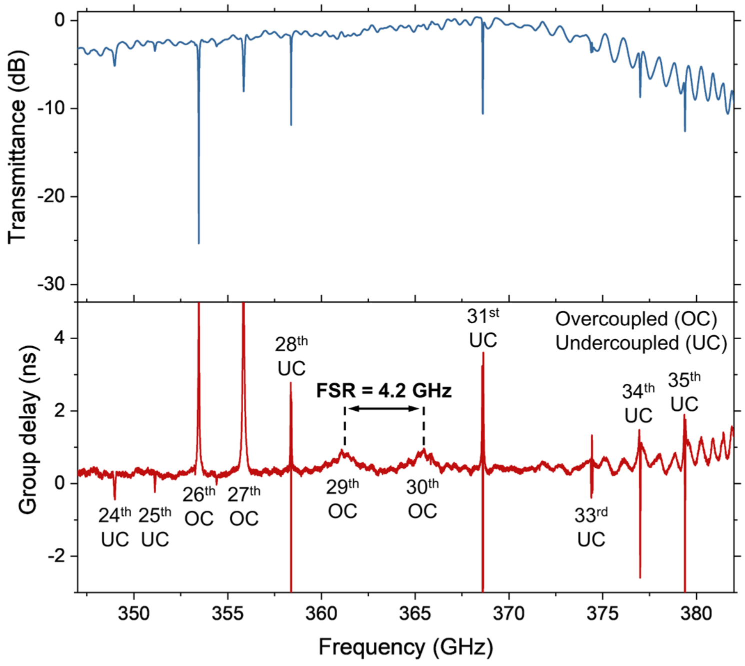

Fig. S7 | Measured transmittance and group delay spectra of the TESC chip with $\Delta L = 0.3a$ and $L = 32a$.

S8. Comparison of TESCs with existing topological cavities

Table S3 summarizes the Q factor and FSR of existing valley-Hall topological cavities. Notably, the simulated Q factor of 219,520 in our TESC is an order of magnitude higher than that of previously reported VPC topological cavities[2], and our measured Q factor of 17,424 is two times higher than the highest measured Q factor of existing topological cavities[3]. In addition, the FSR of our TESC, normalized to the wavelength λ as FSR/λ, is 1.9 times higher than the highest reported value in existing topological cavities[2]. Even when accounting for cavity size, the normalized FSR, FSR$\cdot L/\lambda^2$, of our TESC remains among the highest reported. Our work not only uncovers the physical mechanism underlying the emergence of topological cavity modes but also provides a general and practical method to simultaneously enhance both Q factor and FSR through topological edge state engineering.

Table S3. Comparison of TESCs with existing topological cavities

	Sim. / Exp.	Wavelength (λ)	Q factor	FSR/λ	Side length (L/λ)	FSR*L/λ^2
Ref. [2]	Sim.	1480 nm	21,000	1.0 %	9.8	9.8 %
Ref. [3]	Exp.	1185 nm	8,000	0.06%	6.2	0.37 %
Ref. [4]	Sim.	1490 nm	4,300	-	2.4	-
Ref. [5]	Exp.	1520 nm	1,050	0.7%	9.4	6.58 %
Ref. [6]	Exp.	1536 nm	344	-	5.2	-
**TESC**	Sim.	810 μm	219,520	1.9 %	5.4	10.26 %
	Exp.	837 μm	17,424	1.2 %	8.4	10.08 %

S9. Robustness of TESCs against structural disorders

In this section, we study the effect of fabrication imperfection induced disorder on the resonant frequency and Q_{in} of TESC. The structure is patterned such that each triangular air hole has a random disorder in the side lengths (Fig. S8a). The sizes of all the triangles follow a normal distribution with a standard deviation of 3 μm (Fig. S8b), which is comparable to the fabrication tolerance in the lithography process. The simulation was repeated 10 times

with randomly generated patterns. As shown in Fig. 9, the average Q_{in} of $TE^{18}_{C_{3v},A_1}$ is 87,418, which is 40% of the Q_{in} (219,520) of TESC without disorder for $L = 20a$ and $\Delta L = 0.2a$. The average frequency is 369.75 GHz with a standard deviation of 0.098 GHz, which is very close to 369.61 GHz of the TESC without disorder. For $\Delta L = 0.3a$, the average Q_{in} and frequency are 37,242 and 374.77 GHz, respectively. They also remain close to the Q_{in} (68,065) and frequency (374.76 GHz) of TESC without disorder, demonstrating the robustness of TESCs.

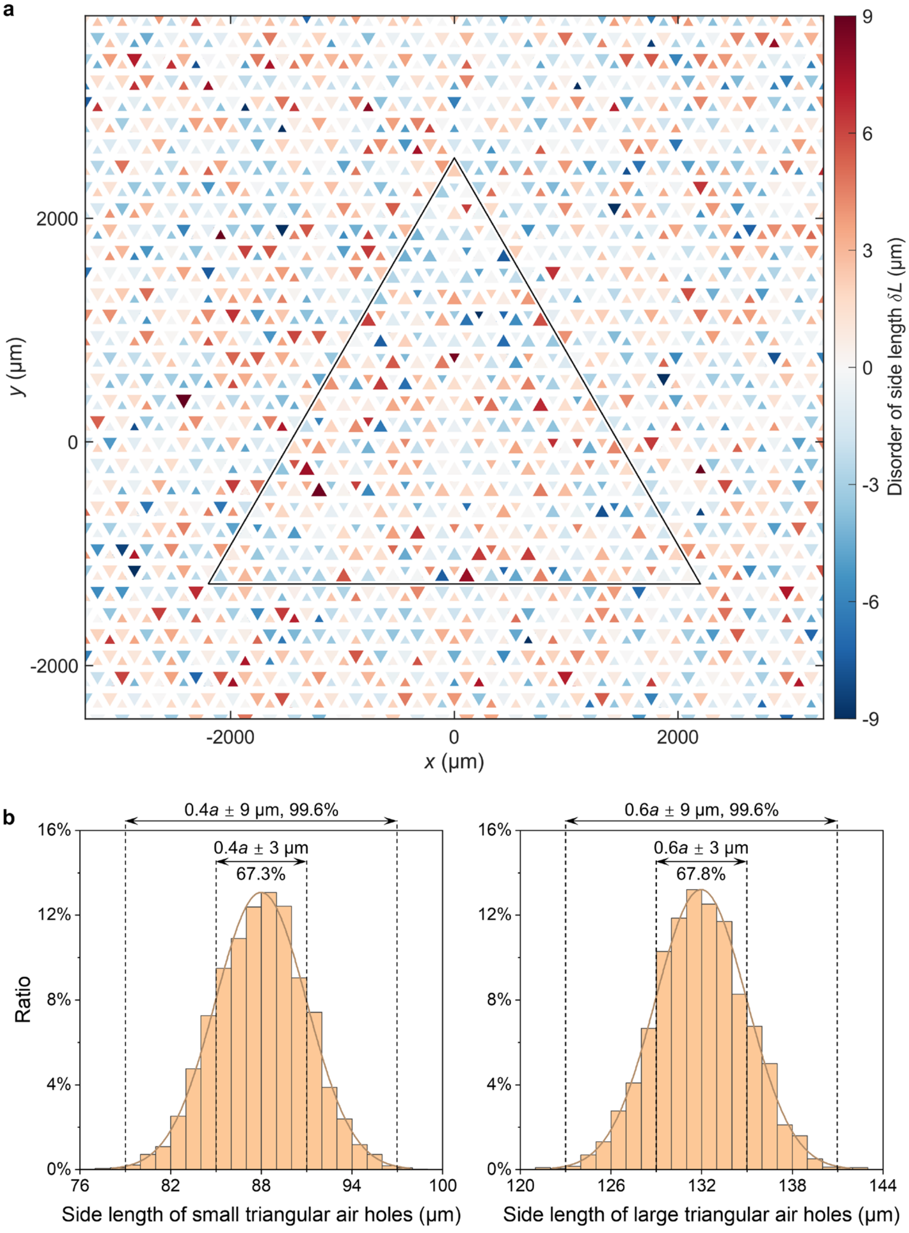

Fig. S8 | TESC with structural disorder. a, The design of a TESC with disorder. The side length is $L = 20a$ and $\Delta L = 0.2a$. Each triangular air hole has a random disorder in the side lengths, with the side length difference δL indicated by the color. **b**, The distribution of the side lengths of small and large triangular air holes in the disordered TESC shown in **a**. They follow a normal distribution with a standard deviation of 3 µm, which is comparable to the fabrication tolerance in the lithography process.

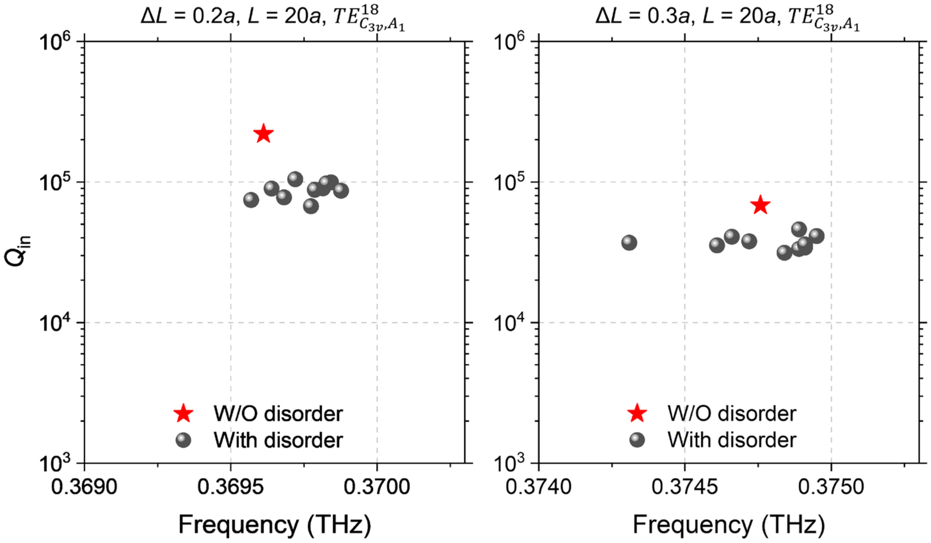

Fig. S9 | Robust Q_{in} and frequency of TESC. Simulated Q_{in} and frequency of TESCs with and without disorder.

S10. Effect of material loss on the *Q* factor of TESC

Fig. S10 shows the calculated Q_{in} of $TE^{18}_{C_{3v},A_1}$ mode for the TESCs with varying material losses, characterized by the loss tangent tan($\varepsilon''/\varepsilon'$), where ε' and ε'' are the real and imaginary parts of the permittivity of silicon, respectively. When the loss tangent is 1×10^{-6}, the Q_{in} is 184,637 which is close to that of the TESC without material loss. It indicates that the radiation loss plays a major role affecting Q_{in} for loss tangent $\leq 1\times10^{-6}$. However, when the loss tangent increases to 1×10^{-5}, which is comparable to that of high-resistivity silicon wafer[7], the Q_{in} decreases to 72,919. As the loss tangent further increases to 1×10^{-4}, Q_{in} decreases significantly to 10,341, indicating that material loss becomes the dominant limiting factor.

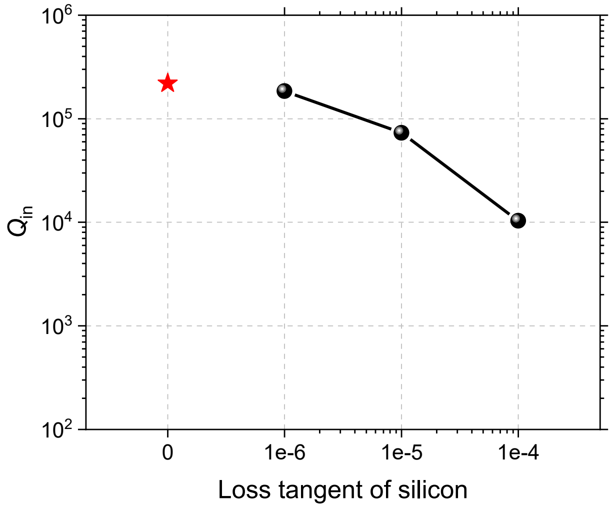

Fig. S10 | Calculated Q_{in} of $\mathrm{TE}^{18}_{C_{3v},A_1}$ of the TESCs with different loss tangents, tan($\varepsilon''/\varepsilon'$), where ε' and ε'' are the real and imaginary parts of the permittivity of silicon, respectively.

S11. Comparison of TESC with WGM and conventional photonic crystal (PC) cavities

Table S4. Comparison of TESC with WGM and conventional PC cavities

	**TESC**	**WGM cavity**	**PC cavity**
Q factor	Both leaky and high-*Q* guided TESCs can be achieved through topological edge state engineering	High *Q* factor can be achieved using large cavity size	Careful design and high-precision fabrication are necessary to achieve high *Q* factor
FSR	FSR can be easily enhanced while maintaining high *Q* factors through topological edge state engineering	Limited FSR in high-*Q* WGM with large cavity size	Difficult to engineer FSR precisely
Device footprint	Compact device footprint enabled by the sharp corners of cavity	Large cavity size is necessary to achieve high *Q* factor	Compact
Fabrication	Nano-fabrication with relaxed precision requirements enabled by the robustness of TESCs against structural disorder	Standard nano-fabrication	High-precision fabrication is necessary

Additional references in Supplementary Information

1 Sakoda, K. & Sakoda, K. *Optical properties of photonic crystals*. Vol. 2 (Springer, 2005).

2 Gong, Y. K. et al. Topological insulator laser using valley-Hall photonic crystals. *ACS Photonics* **7**, 2089-2097 (2020).

3 Xie, X. et al. Topological cavity based on slow-light topological edge mode for broadband Purcell enhancement. *Physical Review Applied* **16**, 014036 (2021).

4 Smirnova, D. et al. Room-temperature lasing from nanophotonic topological cavities. *Light: Science & Applications* **9**, 127 (2020).

5 Gu, L. P. et al. A topological photonic ring-resonator for on-chip channel filters. *Journal of Lightwave Technology* **39**, 5069-5073 (2021).

6 Xie, X. R. et al. Harnessing anti-parity-time phase transition in coupled topological photonic valley waveguides. *Advanced Functional Materials* **33**, 2302197 (2023).

7 Dechwechprasit, P. et al. Terahertz disk resonator on a substrateless dielectric waveguide platform. *Optics Letters* **48**, 4685-4688 (2023).
